# Ensuring the QoE-Related Fairness to Reduce the User Abandonment Ratio

**DOI:** 10.3390/s21217050

**Published:** 2021-10-24

**Authors:** Izabela Mazur, Jacek Rak, Krzysztof Nowicki

**Affiliations:** Faculty of Electronics, Telecommunications and Informatics, Gdańsk University of Technology, G. Narutowicza 11/12, 80-233 Gdańsk, Poland; jrak@pg.edu.pl (J.R.); know@pg.edu.pl (K.N.)

**Keywords:** fairness, QoE, QoS, abandonment ratio

## Abstract

Nowadays, it is quite a challenge for app owners to keep users engaged with an app. Currently, the level of user abandonment is one of the key parameters that application owners are interested in. To meet these challenges, we conduct an extended study of a previously proposed solution that significantly reduces the abandonment rate of a given application. The investigated solution is based on the methods of fairness using the QoE and QoS approach. This paper shows that application abandonment ratios can be reduced by using an appropriate approach to fair bandwidth allocation. Adjusting the bandwidth allocation to users, taking into account the quality of the user experience, has a more effective effect on reducing app abandonment ratios than if quality of service is taken into account. This is because the users make the decision to abandon the application based on their feelings rather than technical parameters. In order to effectively reduce application abandonment ratios, a suitable bandwidth allocation algorithm must be used. This paper presents the impact of using different algorithms on the abandonment ratio and compares the popularly used algorithms and the previously proposed bandwidth allocation algorithm.

## 1. Introduction

In recent years, network traffic has increased significantly, which is a direct cause of resource allocation problems. One of the primary resources that require thoughtful allocation is bandwidth. Furthermore, network traffic is expected to continue to grow in the next few years, so the problem of fair network bandwidth allocation and the associated abandonment of applications by users will continue to be an important issue.

In the past, quality of service (QoS) parameters such as packet loss, latency, jitter and bandwidth were used to measure user satisfaction towards a service or application. However, when there are currently so many different devices and applications in a network, requiring completely different technical parameters, such measurement is not unambiguous and is therefore insufficient. Therefore, a need has emerged for another way of measuring a user’s satisfaction with a service based more on the subjective opinion of the user or on the user’s experience of using the service (often referred to as the quality of experience—QoE [1]). Such a measurement characterises the user’s level of satisfaction with a given application or service, taking into account technical parameters and personality or the current user’s impressions. It is an essential factor because, based on the user’s satisfaction, the user decides to abandon or stay with a given application/service [2]. Paper [3] compares the impact of interference, its intensity and temporal dynamics on user engagement in the context of video streaming. However, it did not propose ways to increase engagement or reduce abandonment.

As shown in [4,5], one of the main factors influencing the abandonment ratio is a long application loading time. From this, it can be deduced that the number of abandonments is directly influenced by bandwidth distribution among the end-users.

In this paper, we would like to answer the question: will a corresponding change in the used fairness approach originally based on QoS parameters towards the one based on QoE affect the number of application abandonments by users? To the best of our knowledge, this question has not been answered in the related literature yet.

### 1.1. Fairness Algorithms Based on QoS Parameters

There are certain algorithms used to ensure fairness. The most popular one is the max-min algorithm [6]. It starts with zero resource allocation for all nodes and next tries to increase the assignment of the network link resources to users until the link becomes saturated. The result of the max-min algorithm is, therefore, a full allocation of the resource such that users with fewer resources have obtained the requested capacity [6,7].

Another important algorithm is the proportional fairness algorithm [7]. In this case, the user is allocated a resource proportional to the request made.

### 1.2. Measurement of QoS Fairness

The most popular measure of fairness is the one proposed by Jain in [8]. The highest value of fairness based on this measure occurs when all users receive the same equal resource allocation, or some users receive no allocation, while the rest receive an equal share of resources. In the case of not receiving any resources, the situation is manifestly and intuitively unfair. However, the case of equal resource allocation does not translate to an equal range of user experience because each user has different hardware, which translates into a different quality [9]. Furthermore, the work [10] very bluntly dismisses equal flow as a solution that is unfair. Since the user decides whether to abandon the application based on feelings and subjective measures rather than objective technical parameters, an equal distribution of service quality parameters will not be beneficial to the providers of applications.

### 1.3. QoE Algorithm

To provide certain minimum values of QoE parameters, specific standards have been created. One of the most commonly used standards for delivering video services is DASH (Dynamic Adaptive Streaming over HTTP). It is based on dividing content into a sequence of small files based on the HTTP protocol. Each of these files represents a short fragment of the transmission of playback content, no more than a few seconds. These segments can be transmitted at different bit rates and are then concatenated into a single coherent content. The intention is to minimise the number of content playback interruptions that may occur due to the changing network conditions [11]. The DASH standard is intended to ensure high utilisation of network resources and provide stable quality of service and increase the user sense of QoE, which is extremely important to reduce the risk of application and service abandonment.

One approach to optimising the network resource allocation and quality adaptation that fairly maximises the QoE of users is shown in [12]. However, in that paper, the authors focus only on one type of service, i.e., video spinning.

### 1.4. Measure of QoE Fairness

Concerning QoE-related fairness, the measured subjective perceptions of end-users must be equal for all. One of the few coefficients to measure fairness precisely in the context of QoE is the index proposed in [13] and developed in [14].

(1) $$F=1-\frac{2\sigma }{H-L}$$

where:

(2) $$\sigma^{2}=\frac{1}{n-1}\sum_{i=1}^{n}{\left(Y_{i}-\mu \right)}^{2}$$

(3) $$\mu =\frac{\sum_{i=1}^{n}Y_{i}}{n}$$

where:

*i* is the user index;
$Y_{i}$ is the parameter of QoE;
*L* is the lowest bound of the QoE parameter;
*H* is the upper bound of the QoE parameter;
*n* is the standard deviation of the QoE parameter;
μ is the arithmetic mean of the QoE parameter.

The main difficulty in using this measure is the effective and appropriate mapping of the technical parameters of the network—QoS—to the quality of experience parameters of users—QoE [15].

#### MOS—Mean Opinion Score

The mean opinion score is the most commonly used measure to determine the quality of an experience. It is used to express the quality of a system. It is most often calculated as an arithmetic mean of the collected user opinions. However, this method is time-consuming and expensive. This is due to the need to involve many people to express their opinions. Objective quality methods are also used. These methods are trained based on the use of opinions set by people [16].

In this paper, a 5-degree scale (1–5) is used (see Table 1), as recommended in [17].

The remainder of the paper is organised as follows: Section 2 focuses on the motivation for addressing the topic and outlines why we feel there is a need to address the topic of equity in order to reduce application abandonment. In Section 3, we present the necessary equations describing the relationship between QoS and QoE parameters for selected types of applications. In Section 4, we present our algorithm from [19], here accompanied by an extended set of possible scenarios to be investigated, while, in Section 5, we provide a description of the respective simulation assumptions. The results of the comparison of the investigated algorithm with commonly used methods are presented in Section 6. Section 7 concludes the paper.

## 2. Motivation for Addressing the Topic

Providers and users evaluate the performance of an application. Providers most often use quality-of-service parameters such as throughput, latency or the loss ratio. However, users are less interested in technical parameters and primarily base their opinions on subjective perceptions, i.e., quality of experience (QoE). Users expect good perceptual quality, which can be derived from many factors, including not only technical parameters but also user experience [2].

Current solutions available for fair resource sharing primarily focus on allocating capacity based on QoS parameters only. However, such an approach does not provide adequate QoE values. A strategy that focuses on considering fairness from the QoE perspective guarantees higher overall end-user satisfaction and thus reduces the number of users abandoning the applications.

We present our solution to address the lack of appropriate mechanisms to ensure QoE fairness for different types of applications. In our solution, we divide users into satisfied and unsatisfied users and ensure equal QoE performance among satisfied users, regardless of the application used.

This algorithm mainly targets large subnetworks where users are aware of other users’ experiences, including, e.g., online game championships, where users are aware of the feelings of other players, or large subnetworks such as corporations, student residences or university campuses. In such sub-networks, users of different types of applications co-exist, are in direct contact with other users and are aware of the experience of other users’ application usage.

In our previous paper [19], we proposed a fairness algorithm designed for a limited set of application types. In the current paper, the implementation of the algorithm has been extended to cover a broad range of application types and different arrival times of requests.

## 3. Expected User Opinion

In this paper, we refer to four types of different applications: file downloading, web browsing, VoIP according to G.722 codec and VoIP according to G.726/G.727 codec. The opinion is expressed on a 5-degree MOS scale. We define an unsatisfied user as a user whose quality of experience is low enough to abandon the used application. An opinion equal to 3.0 was arbitrarily chosen as the boundary between the groups of satisfied and unsatisfied users. When the user’s opinion is below 3.0, the user is referred to as unsatisfied (and satisfied otherwise).

### 3.1. Web Browsing

According to the research results described in [20], the relationship between a long session duration and end-user feedback can be expressed as follows:

(4) $$MOS_{i}=5.72-0.936\cdot log\left(session\_time_{i}\right)$$

where *i* is the user number, and session_time is the duration of the user’s web browsing in seconds.

### 3.2. File Downloading

In the case of file downloading, one of the most important parameters for the user’s perception of the quality of experience is bandwidth. Another essential parameter is the file size, which affects the user’s expected download time.

In [21], Equation (5) was provided to represent the user’s opinion depending on the file size and bandwidth:

(5) $$MOS_{i}=\frac{0.755}{\sqrt{f_{i}}}\cdot log\left(bw_{i}\right)+1.268$$

where *i* is the user’s number, *f* is the normalised download size in bits, and $bw_{i}$ is the allocated bandwidth in bps.

### 3.3. VoIP

Paper [22] presents the results of a study on the effect of allocated bandwidth on the user opinion for VoIP applications. The respective VoIP-related measure from [22] is presented in Equation (6).

(6) $$MOS_{i}=a-b\cdot ln\left(\frac{c}{bw-d}\right)$$

where *i* is the user’s number, bw denotes the allocated bandwidth in bps while *a*, *b*, *c* and *d* are generic parameters depending on the codecs used (as paper [22] does not detail the meaning of the individual parameters *a*, *b*, *c* and *d*).

## 4. Methods

### 4.1. Investigated Algorithm

The goal of the algorithm is to distribute bandwidth fairly in terms of QoE for satisfied users and fairly in terms of QoS for unsatisfied users. Our algorithm is based on dividing users into satisfied and unsatisfied based on the predicted final user opinion. The final opinion is predicted based on the developed patterns tailored to the type of application used by the user. As an input, the user provides the desired bandwidth size, the type of application being used and the parameters needed to support the application. In the case of downloading files, it is the size of the file; in the case of web surfing, it is the size of the web page; in the case of VoIP services, it is the duration of the service.

**Figure 1:**
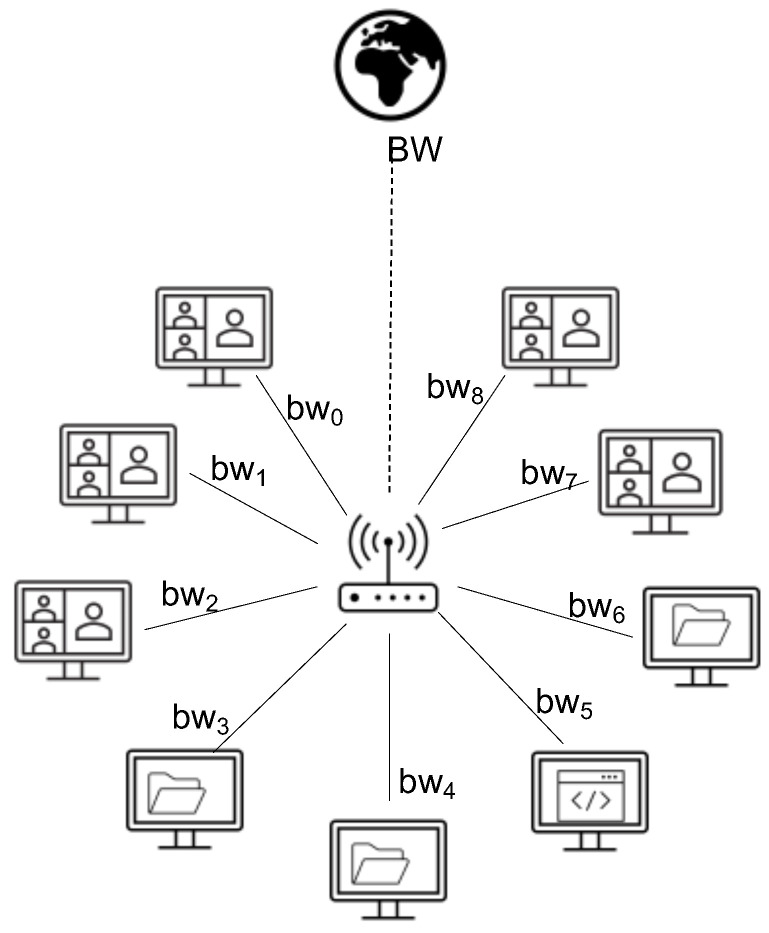
The topology used in this paper for investigation of the fairness schemes.

**Figure 2:**
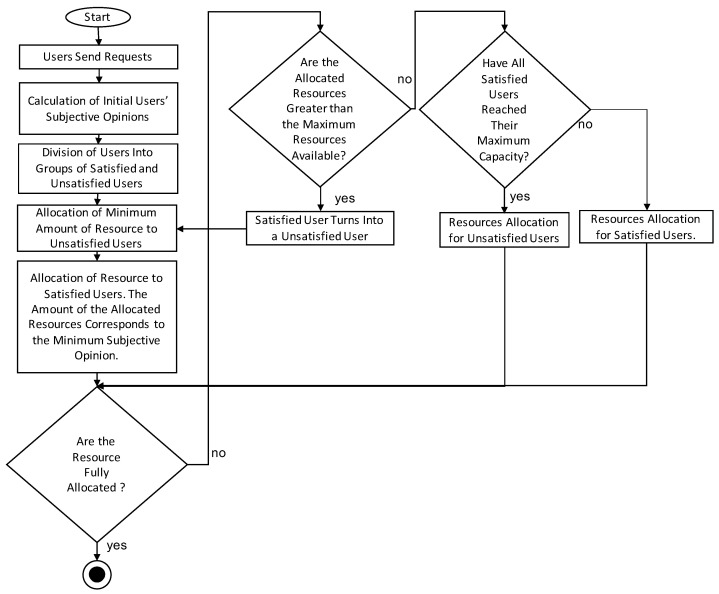
Scheme of the algorithm.

We use a star topology in this paper. In Figure 1, $bw_{i}$ refers to the maximum bandwidth of user *i*, while BW refers to the outgoing link capacity. The entire algorithm is presented in Figure 2.

### 4.2. Consecutive Steps of Our Algorithm

*Users Send Requests*: The user sends a request to the computing unit. The request contains information such as the maximum possible bandwidth for a given user, type of served application and other parameters such as the size of the downloaded file (in the case of file downloading) or the size of the webpage (in the case of web browsing).

*Calculation of Initial Users’ Subjective Opinions*: The computing unit calculates the preliminary values of the subjective opinion of the users based on the considered applications.

*Division of Users into Groups of Satisfied and Unsatisfied Users*: Based on opinions calculated in the previous stage, users are divided into those who are satisfied and those who are unsatisfied.

*Allocation of Minimal Amount of Resources to Unsatisfied Users*: Unsatisfied users are allocated a minimum bandwidth, following the approach from [19], as defined by Equation (7):
  (7) $$bwTemp_{l}=q\cdot bwTemp_{minUnsatisfied}$$
  where:
  *l*—index of the unsatisfied user (l=1,2,3…,k);
  *q*—service provider’s coefficient *q* ∈(0,1>;
  $bwTemp_{minUnsatisfied}$ =$min(bw_{min};\frac{BW}{n})$;
  $bw_{min}$—minimum requested bandwidth among unsatisfied users.

*Allocation of Resources to Satisfied Users—The Amount of Allocated Resources Corresponds to the Minimum Subjective Opinion*: The bandwidth is allocated to all satisfied users according to the equation in accordance with using the application (Equations (4), (5) or (6)).

*Are the Resources Fully Allocated?*: This step is to verify whether the amount of allocated resources for satisfied and unsatisfied users sums up to the maximum available amount.

*Are the Allocated Resources Greater than the Maximum Resource Available?*: The purpose of this step is to verify if the amount of allocated resources for satisfied and unsatisfied users is greater than the maximum amount of available resources.

*Satisfied User Turns into an Unsatisfied User*: If the amount of allocated resources for satisfied and unsatisfied users is greater than the total amount of available resources, it is necessary to move one user who is satisfied to unsatisfied to release the allocated bandwidth. In order to release as much of the resources as possible at the lowest possible cost concerning the increase in the number of unsatisfied users, it is necessary to apply this change to the user who requires the largest amount of resources.

*Have All Satisfied Users Reached Their Maximum Capacity?*: This step is to check if all satisfied users have reached their maximum capacity.

*Resource Allocation for Satisfied Users*: If not all satisfied users have reached their maximum bandwidth, bandwidth is allocated to satisfied users with the minimum subjective opinion increase.

*Resource Allocation for Unsatisfied Users*: Suppose that the bandwidth is distributed according to all the previous steps and the amount of bandwidth allocated to all users is less than the total available outgoing link capacity, and all satisfied users have reached their maximum bandwidth. In this case, the remaining part of the bandwidth is distributed among unsatisfied users. It is worth noting that this operation will not change the status of unsatisfied users to satisfied users but is intended to maximise the usage of the entire network’s resources.

### 4.3. Example of Use Case of the Investigated Algorithm

**Figure 3:**
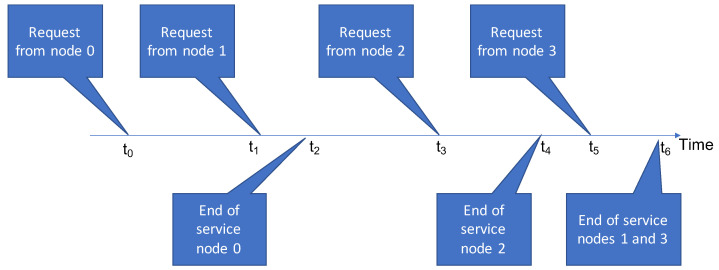
Example of use case of the investigated algorithm.

An example of using the algorithm is shown in Figure 3. At time $t_{0}$, user $n_{0}$ makes a request to utilise a given application. At time $t_{0}$, the bandwidth allocated to this application is calculated according to the investigated algorithm. Then, at time $t_{1}$, another request comes—this time from user $n_{1}$. Again, the bandwidth allocation for users $n_{0}$ and $n_{1}$ is calculated. At time $t_{2}$, user $n_{0}$ stops using the application and, once again, the bandwidth allocation is re-calculated for the user who continues to use the application.

## 5. Algorithm Assumptions

As mentioned in Section 3.1, in this paper, we focus on four types of applications: file downloading, web browsing, VoIP according to G.722 codec and VoIP according to G.726/G.727 codec. The limiting MOS value classifying users into satisfied and unsatisfied was arbitrarily set to 3.0. Simulations were carried out for a outgoing link capacity equal to 500 Mbps and for different numbers of users. The number of users ranged between 10 and 750. Only in the case of the analysis for application distribution scenario 3 (see Table 2), where users were only using web surfing, the outgoing link capacity was 10Mbps. This was due to the fact that application distribution scenario 3 was not very demanding in terms of desired bandwidths and had a short execution time. To model the network congestion scenario, we reduced the outgoing link capacity to 10 Mbps.

In order to use Equation (6), it was necessary to determine the values of parameters *a*, *b*, *c* and *d*. This is because, when trying to reproduce the graph from the paper [22], it was noticed that, after substituting given parameter values, it was not possible to obtain the same graph. However, in order not to abandon the data presented in the paper, the measurement points presented in the graphs were used in [22]. Based on these points, coefficients matching Equation (6) were selected as presented in Table 3 to obtain a function similar to that presented in [22].

## 6. Results

**Figure 4:**
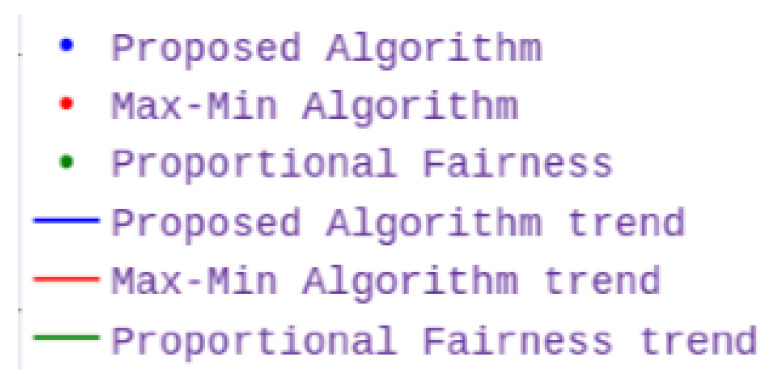
The legend valid for all figures in the remaining part of the paper.

Simulations were performed to analyse the number of unsatisfied users after applying three bandwidth allocation algorithms: the max-min algorithm, the proportional fairness scheme and our algorithm. Compared to the two common algorithms, the number of unsatisfied users was lower or equal in all the analysed cases after applying the investigated algorithm. All figures shown use the same symbol structure shown in Figure 4.

All considered scenarios are presented in Table 2. The greatest advantage of the investigated algorithm was seen in scenarios of high network congestion. In general, as shown later in this paper, as the network load increases, the benefits of the investigated algorithm increase as well.

### 6.1. Relationship between the Number of Users and Application Abandonment Ratio

**Figure 5:**
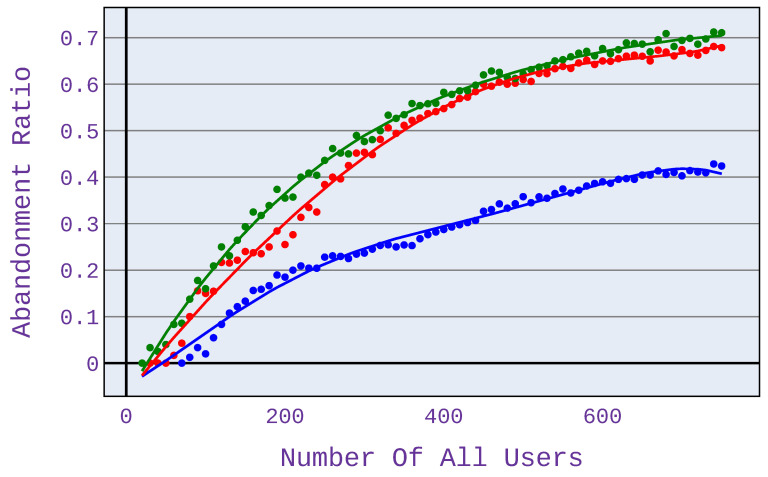
Abandonment ratio related to the number of users for application distribution scenario 1.

When users use different types of applications, as presented in Figure 5 (25% of users download files, 25% of users surf the web, 25% use VoIP services following G.726 or G.727 codecs and 25% use VoIP services following G.722 codecs), the benefits of the algorithm become visible already for 50 users. Moreover, as the number of users increases (and thus the size of the network load, too), these benefits increase. At approximately 400 users, our algorithm’s advantage in terms of reducing the number of unsatisfied users (compared to conventional schemes) reaches approximately 25%. It persists up to the maximum load calculated in this simulation.

**Figure 6:**
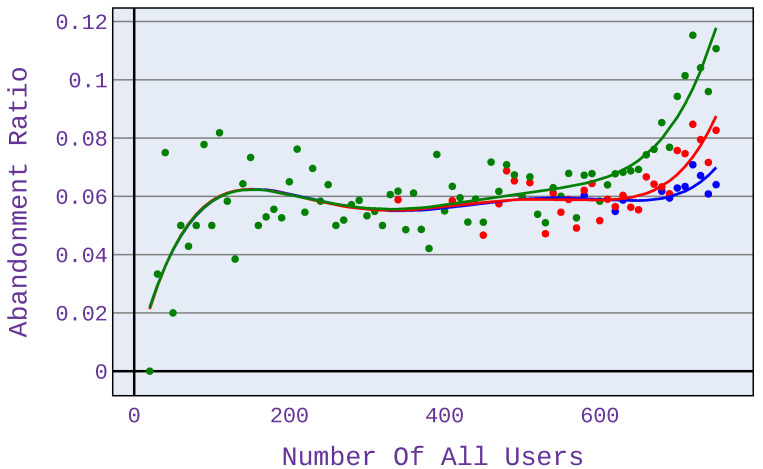
Abandonment ratio related to the number of users for application distribution scenario 2.

**Figure 7:**
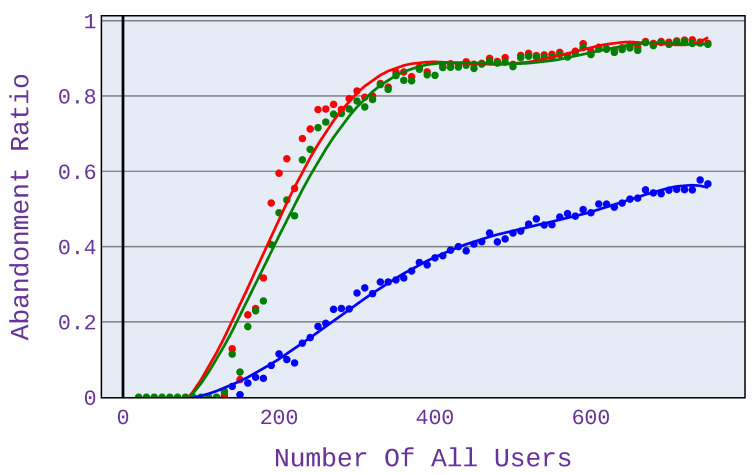
Abandonment ratio related to the number of users for application distribution scenario 3.

**Figure 8:**
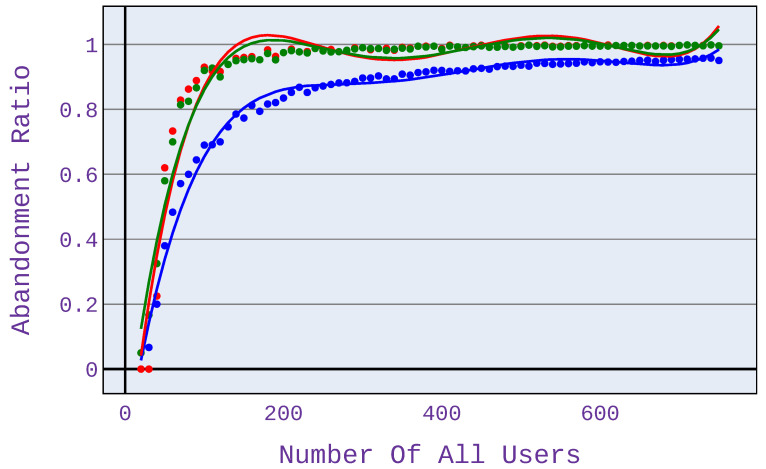
Abandonment ratio related to the number of users for application distribution scenario 4.

**Figure 9:**
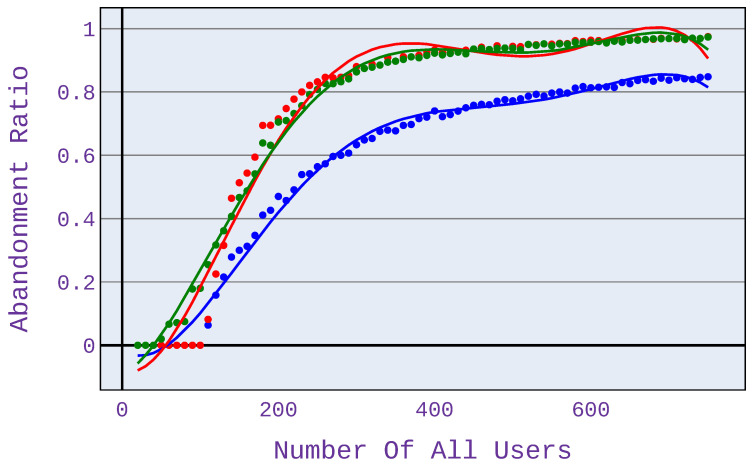
Abandonment ratio related to the number of users for application distribution scenario 5.

In case scenario 2 (Figure 6), when users only download files, the benefits are visible at high network load—there are at least 700 users served. This is because, with fewer users, network congestion does not occur, so mechanisms to limit the allocated bandwidth are not triggered. It is important to note that the different types of applications shown in Figure 6, Figure 7, Figure 8 and Figure 9 have different points of network congestion, and therefore a different trigger point for the mechanism responsible for limited bandwidth allocation to users.

Significant benefits from using our approach can be seen when users only browse the web (see Figure 7). The profit, in this case, reaches up to almost 60% (for 300 users). Application providers based on web browsing can benefit the most from the investigated algorithm because, here, the benefits of the investigated algorithm are the greatest.

In Figure 8 and Figure 9, it can be seen that the magnitude of the benefit of the investigated algorithm depends on the codecs used when using a VoIP service. For G.726/G.727 codecs, the benefits are less than for G.722 codecs.

For the computations presented in this paper, we use the max-min and proportional fairness algorithms based on the most commonly used method, which is the partitioning with regard to bandwidth. However, for the algorithm under study, the allocation is based on the predicted MOS opinion. This approach helps us to immediately select users who will not be satisfied and thus not invest in them and be able to focus our attention on users who are potentially satisfied. In the case of algorithms such as max-min or proportional fairness, which are based only on bandwidth, they are not able to determine the users who are worth dropping and thus assign them a higher value of bandwidth that they could assign to other users who might be satisfied with this additional value of bandwidth. Therefore, the results concerning the number of satisfied users obtained by our approach are visibly better than the respective ones for the reference max-min scheme (see, e.g., Figure 7 presenting the advantage of up to 60%).

This approach is thus beneficial from the viewpoint of service providers and application owners who compete for every user and who have no advantage in investing in users who abandon their application/service.

### 6.2. Relationship between the Number of Users and Fairness of Satisfied Users

In order to compare fairness values, we also present the user satisfaction factor of the fairness measure—this is because comparing only fairness values does not show the clear benefit of the investigated algorithm. The strength of the investigated algorithm is precisely to minimise the abandonment rate and thus to maximise the number of satisfied users. Therefore, it was decided to modify the fairness measure, as presented in Equation (8).

(8) $$F_{satisfied}=F_{H}\cdot m/n$$

where: $F_{H}$—fairness index based on [13], *m*—number of satisfied users, *n*—number of all users.

**Figure 10:**
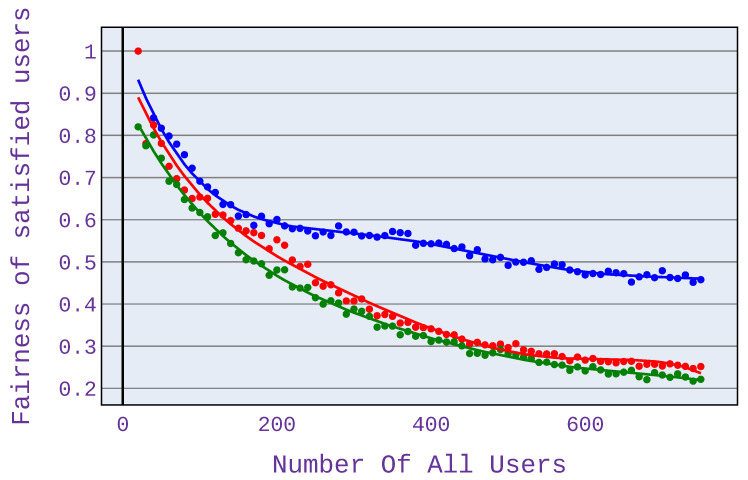
Fairness index for application distribution scenario 1.

As can be seen in Figure 10, the highest value of the fairness index is achieved for application distribution scenario 1, which means an equal share of different application types in the simulation. This is because, for this scenario, there is the most significant difference in terms of the number of satisfied users among the algorithms used.

**Figure 11:**
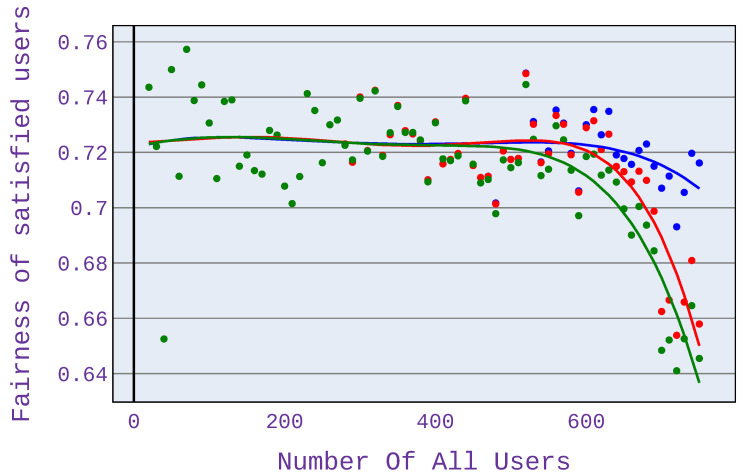
Fairness index for application distribution scenario 2.

As with the abandonment rate analysis for Scenario 2 shown in Figure 11, higher fairness index values occur with more users. As before, this is due to the occurrence of a network congestion point.

**Figure 12:**
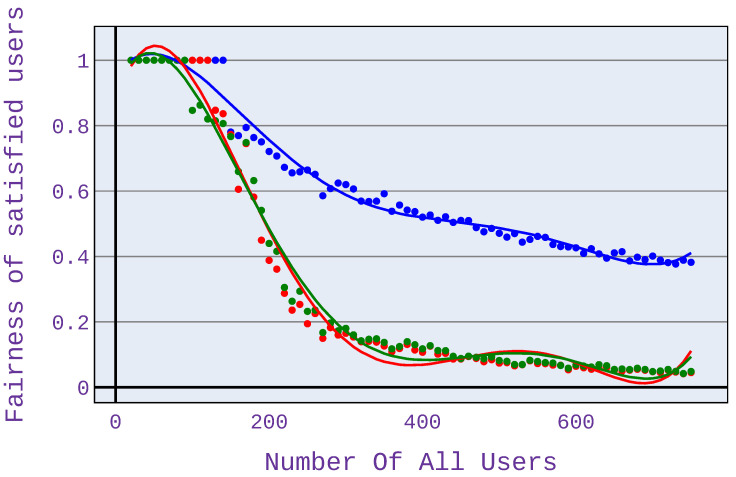
Fairness index for application distribution scenario 3.

A significant difference in the fairness index can also be seen when using only web browsing (Figure 12). The fairness is then higher even by 40%.

Our experiments have also shown that when using only VoIP applications, the differences are not so remarkable, although there is a visible gain.

### 6.3. Impact of the Chosen Level of Minimum MOS

The minimum MOS value, which represents the boundary between satisfied and unsatisfied users, has been set to 3.0 in all simulations. However, this is a value that can be adjusted as needed. In the following section, we show how the abandonment rate behaves depending on the set minimum MOS.

**Figure 13:**
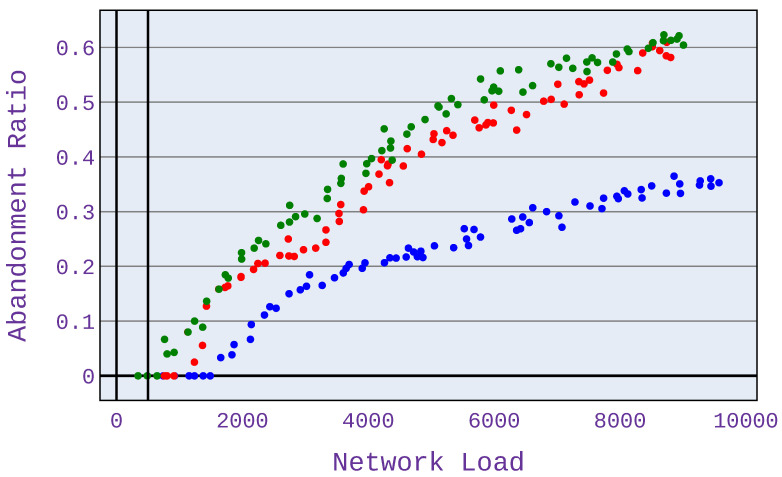
Abandonment ratio related to mean network load (Mbps) for application distribution scenario 1. Minimum MOS set to 2.0.

**Figure 14:**
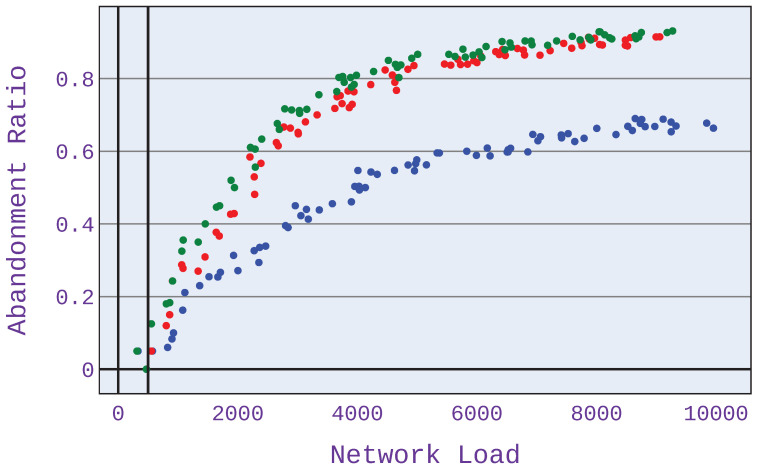
Abandonment ratio related to mean network load (Mbps) for application distribution scenario 1. Minimum MOS set to 4.0.

It can be seen in Figure 13 and Figure 14 that for a lower value of MOS, the abandonment ratio is lower. This is due to the lower expectations of users regarding the bandwidth allocated, allowing more users to be served at a level that satisfies them. However, controlling the MOS value should be based on actual data from users. For this purpose, it is necessary to collect information from users at the level of satisfaction on a 5-degree scale that they are satisfied with for a given application. The algorithm can be adjusted so that, for each application distribution scenario, the minimum MOS value is configured at a different level.

## 7. Conclusions

As presented in this paper, the use of equity mechanisms based on the quality of experience has a positive effect on reducing abandonment ratios, which are often high for QoS-related mechanisms. In each of the examples provided, after applying the investigated algorithm, the abandonment ratio was lower or equal after applying popular algorithms. It is highly beneficial for application providers interested in the lowest abandonment ratio and the highest number of users utilizing the applications. It is especially noticeable in the case of scenarios 1 and 3, where the differences in the size of the abandonment rate between the applied algorithms are the most significant. It should be noted that the most significant benefit of the investigated algorithm is in situations of high network congestion.

The main difficulty in applying our algorithm is the need to determine the QoS-to-QoE mapping function. Due to difficulties in performing such a study, there are only a few mapping functions available for different types of applications. This, in turn, limits the applicability of our algorithm only to applications for which such a function has been determined.

As future work, a comprehensive study on the mapping of QoS parameters to QoE is planned for different types of applications. Ideally, this research should be carried out for a significant number of users drawn from different groups to obtain adequate results for all users, not merely for a particular group.

## Figures and Tables

**Table 1:** Five-grade MOS scale from [18].

Opinion	Label
5	Excellent
4	Good
3	Satisfactory
2	Poor
1	Bad

**Table 2:** Analysed scenarios of distribution of users referring to the considered applications [%].

Application Distribution	File	Web	VoIP Codecs	VoIP Codecs
Scenario	Downloading	Surfing	G.726 and G.727	G.722
1	25	25	25	25
2	100	0	0	0
3	0	100	0	0
4	0	0	100	0
5	0	0	0	100
6	50	50	0	0
7	50	0	50	0
8	50	0	0	50
9	0	50	50	0
10	0	50	0	50
11	0	0	50	50
12	0	33	33	33
13	33	0	33	33
14	33	33	0	33
15	33	33	33	0
16	25	75	0	0
17	75	25	0	0
18	25	0	75	0
19	75	0	25	0
20	25	0	0	75
21	75	0	0	25
22	0	25	75	0
23	0	75	25	0
24	0	25	0	75
25	0	75	0	25
26	0	0	25	75
27	0	0	75	25

**Table 3:** Fitting parameters of the general equation based on values from [22] for some codecs.

Codecs	a	b	c	d
G.726 and G.727	1.3557	0.8952	0.7795	13.8186
G.722	1.8778	0.6207	0.2216	4.3825

## Data Availability

The data presented in this study are available on request from the corresponding author.
